# Climatic and geographic predictors of life history variation in Eastern Massasauga (*Sistrurus catenatus*): A range-wide synthesis

**DOI:** 10.1371/journal.pone.0172011

**Published:** 2017-02-14

**Authors:** Eric T. Hileman, Richard B. King, John M. Adamski, Thomas G. Anton, Robyn L. Bailey, Sarah J. Baker, Nickolas D. Bieser, Thomas A. Bell, Kristin M. Bissell, Danielle R. Bradke, Henry Campa, Gary S. Casper, Karen Cedar, Matthew D. Cross, Brett A. DeGregorio, Michael J. Dreslik, Lisa J. Faust, Daniel S. Harvey, Robert W. Hay, Benjamin C. Jellen, Brent D. Johnson, Glenn Johnson, Brooke D. Kiel, Bruce A. Kingsbury, Matthew J. Kowalski, Yu Man Lee, Andrew M. Lentini, John C. Marshall, David Mauger, Jennifer A. Moore, Rori A. Paloski, Christopher A. Phillips, Paul D. Pratt, Thomas Preney, Kent A. Prior, Andrew Promaine, Michael Redmer, Howard K. Reinert, Jeremy D. Rouse, Kevin T. Shoemaker, Scott Sutton, Terry J. VanDeWalle, Patrick J. Weatherhead, Doug Wynn, Anne Yagi

**Affiliations:** 1 Department of Biological Sciences, Northern Illinois University, DeKalb, Illinois, United States of America; 2 Seneca Park Zoo, Rochester, New York, United States of America; 3 Department of Zoology, The Field Museum, Chicago, Illinois, United States of America; 4 Cornell Lab of Ornithology, Cornell University, Ithaca, New York, United States of America; 5 Illinois Natural History Survey, Prairie Research Institute, University of Illinois at Urbana, Champaign, Champaign, Illinois, United States of America; 6 Department of Biology, Indiana University-Purdue University Fort Wayne, Fort Wayne, Indiana, United States of America; 7 New York State Department of Environmental Conservation, Albany, New York, United States of America; 8 Michigan Department of Natural Resources, Wildlife Division, Grass Lake, Michigan, United States of America; 9 Department of Biology, Grand Valley State University, Allendale, Michigan, United States of America; 10 Department of Fisheries and Wildlife, Michigan State University, East Lansing, Michigan, United States of America; 11 University of Wisconsin, Milwaukee, Field Station, Saukville, Wisconsin, United States of America; 12 Ojibway Nature Centre, City of Windsor, Windsor, Ontario, Canada; 13 Department of Biological Sciences, Bowling Green State University, Bowling Green, Ohio, United States of America; 14 Department of Natural Resources and Environmental Sciences, University of Illinois, Urbana, Illinois, United States of America; 15 Alexander Center for Applied Population Biology, Lincoln Park Zoo, Chicago, Illinois, United States of America; 16 Turtles for Tomorrow, Madison, Wisconsin, United States of America; 17 Urban Chestnut Brewing Company, St. Louis, Missouri, United States of America; 18 Department of Environmental and Forest Biology, State University of New York College of Environmental Science and Forestry, Syracuse, New York, United States of America; 19 Department of Biology, State University of New York Potsdam, Potsdam, New York, United States of America; 20 Chesapeake Bay Foundation, Richmond, Virginia, United States of America; 21 Michigan Natural Features Inventory, Michigan State University Extension, Lansing, Michigan, United States of America; 22 Toronto Zoo, Scarborough, Ontario, Canada; 23 Forest Preserve District of Lake County, Libertyville, Illinois, United States of America; 24 Bureau of Natural Heritage Conservation, Wisconsin Department of Natural Resources, Madison, Wisconsin, United States of America; 25 Ojibway Nature Centre, City of Windsor, Windsor, Ontario, Canada; 26 Parks Canada, Gatineau, Québec, Canada; 27 Parks Canada, Midland, Ontario, Canada; 28 United States Fish & Wildlife Service, Chicago, Illinois, United States of America; 29 Department of Biology, The College of New Jersey, Ewing, New Jersey, United States of America; 30 Ontario Ministry of Natural Resources, Parry Sound, Ontario, Canada; 31 Department of Natural Resources and Environmental Science, University of Nevada, Reno, Nevada, United States of America; 32 Stantec Consulting Services Inc, Independence, Iowa, United States of America; 33 Department of Natural Resources and Environmental Sciences, University of Illinois, Urbana, Illinois, United States of America; 34 Department of Evolution, Ecology, and Organismal Biology, The Ohio State University, Columbus, Ohio, United States of America; 35 Ministry of Natural Resources, Vineland Station, Ontario, Canada; University of Regina, CANADA

## Abstract

Elucidating how life history traits vary geographically is important to understanding variation in population dynamics. Because many aspects of ectotherm life history are climate-dependent, geographic variation in climate is expected to have a large impact on population dynamics through effects on annual survival, body size, growth rate, age at first reproduction, size–fecundity relationship, and reproductive frequency. The Eastern Massasauga (*Sistrurus catenatus*) is a small, imperiled North American rattlesnake with a distribution centered on the Great Lakes region, where lake effects strongly influence local conditions. To address Eastern Massasauga life history data gaps, we compiled data from 47 study sites representing 38 counties across the range. We used multimodel inference and general linear models with geographic coordinates and annual climate normals as explanatory variables to clarify patterns of variation in life history traits. We found strong evidence for geographic variation in six of nine life history variables. Adult female snout-vent length and neonate mass increased with increasing mean annual precipitation. Litter size decreased with increasing mean temperature, and the size–fecundity relationship and growth prior to first hibernation both increased with increasing latitude. The proportion of gravid females also increased with increasing latitude, but this relationship may be the result of geographically varying detection bias. Our results provide insights into ectotherm life history variation and fill critical data gaps, which will inform Eastern Massasauga conservation efforts by improving biological realism for models of population viability and climate change.

## Introduction

Knowledge of how life history traits vary geographically is important to understanding variation in population dynamics and improving biological realism for models of population viability and response to climate change [1–3]. Phenotypic variation may be a consequence of developmental plasticity, heritable differences within and among populations, or interplay between these factors [4]. Additionally, biotic and climate gradients jointly regulate variation in resource availability and thus help shape life histories [5]. Consequently, selective pressures imposed by local conditions can result in life history variation among populations. This variation may be especially evident in species with broad geographic distributions (e.g. [6–8]). However, heterogeneity among populations can also occur within small geographic areas when fine-scale climate (e.g., temperature, precipitation) and environmental factors (e.g., competition, prey availability, habitat composition and structure) vary sharply across the landscape [9–11].

The Eastern Massasauga (*Sistrurus catenatus*) is a small, secretive rattlesnake with a distribution centered on the Great Lakes region of North America. Although historically considered subspecies, multilocus DNA sequence data demonstrate that the Eastern Massasauga is taxonomically distinct from the Western Massasauga (*S*. *tergeminus*) [12, 13]. Local weather and climate within the Eastern Massasauga’s range are strongly influenced by lake effects [14]. Many aspects of ectotherm life history are climate dependent ([15, 16], reviewed in [4, 17, 18]). Thus, geographic variation in climate is expected to have a large impact on Eastern Massasauga population dynamics through effects on annual survival, body size, growth rate, age at first reproduction, reproductive frequency, and fecundity. Consistent with this expectation, annual adult survival for *S*. *catenatus* increases along a southwest to northeast axis, ranging from 0.35 to 0.95 (see Fig 3 in [7]). Similarly, litter size shows a two‐fold increase from 6 to 12 offspring from southern to northern sites (see Fig 1 in [19]). However, it is unknown whether latitudinal litter size patterns result from females being larger at northern sites, from a change in litter-size/female-size relationships, or both.

The Eastern Massasauga is considered threatened or endangered everywhere it occurs and was listed under the Endangered Species Act in the United States in 2016 [20, 21]. As a consequence, extinction risk for this species has been extensively modeled using a range of parameter estimates and approaches [7, 22–28]. Unfortunately, such conservation evaluations are hampered by data gaps in life history traits (e.g., growth rate, age at first reproduction, body size, reproductive frequency, and size-specific fecundity) and demography (e.g., population size). Consequently, modelers have often substituted estimates from distant populations or relied upon expert opinion to fill these gaps.

The 2016 federal listing has increased the need to improve biological realism for extinction risk models (e.g., [28]). An obvious strategy for improving model realism and the predictive power of these models is to incorporate estimates of life history parameters and associated variances, which are still lacking for many populations. Fortunately, the Eastern Massasauga has been the focus of research throughout much of its range. As a result, detailed, site‐specific information on Eastern Massasauga life history exists but has not been synthesized in a systematic way. The purpose of this study is to fill existing data gaps in Eastern Massasauga life history. Therefore, our specific objectives are to 1) synthesize available life history data and describe range-wide patterns of variation in these traits, 2) elucidate the abiotic factors that best explain this variation, and 3) gain insight into processes that may have given rise to these geographic patterns.

## Materials and methods

**Fig 1:**
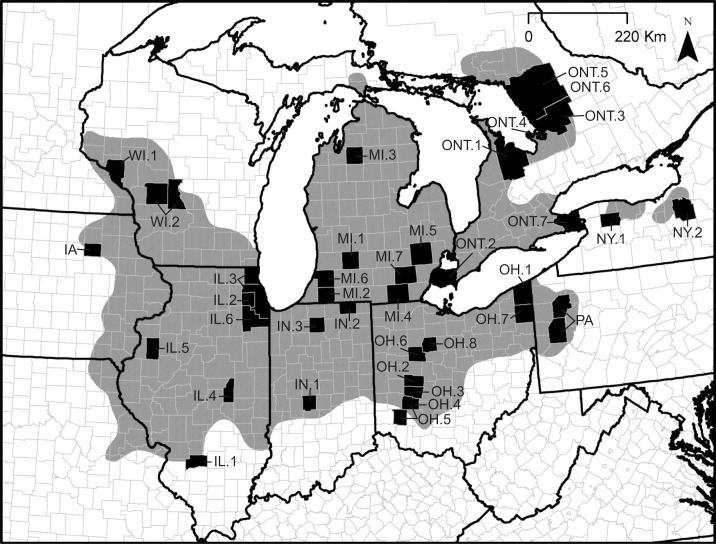
Locations of Eastern Massasauga study sites (counties/districts shaded black) and the approximate historic range of the Eastern Massasauga (gray shading, from http://www.iucnredlist.org/). County and district codes: IA = Bremer, IA; IL.1 = Clinton, IL; IL.2 = DuPage, IL; IL.3 = Cook/ Lake, IL; IL.4 = Piatt, IL; IL.5 = Warren, IL, IL.6 = Will, IL; IN.1 = Hendricks, IN; IN.2 = LaGrange, IN; IN.3 = Marshall, IN; MI.1 = Barry, MI; MI.2 = Cass, MI; MI.3 = Kalkaska, MI; MI.4 = Lenawee, MI; MI.5 = Oakland, MI; MI.6 = Van Buren, MI; MI.7 = Washtenaw, MI; NY.1 = Genesee, NY; NY.2 = Onondaga, NY; OH.1 = Ashtabula, OH; OH.2 = Champaign, OH; OH.3 = Clark, OH; OH.4 = Greene, OH; OH.5 = Greene/ Warren, OH; OH.6 = Hardin, OH; OH.7 = Trumball, OH; OH.8 = Wyandot, OH; ONT.1 = Bruce, ONT; ONT.2 = Essex, ONT; ONT.3 = Muskoka, ONT; ONT.4 = Beausoliel Island, ONT; ONT.5 = Parry Sound District (1995–1996), ONT; ONT.6 = Parry Sound District (1992–2009), ONT; ONT.7 = Regional Municipality of Niagara, ONT; PA = Butler/ Venango, PA; WI.1 = Buffalo, WI; WI.2 = Juneau/ Monroe, WI. Reprinted and modified from [150] under a CC BY license, with permission from [Collin P. Jaeger], original copyright [2016] (See S3 File).

We compiled life history information collected by researchers over the past 128 years (1886–2014) from peer-reviewed publications, technical reports, and collaborators for 47 study sites representing 38 counties in nine North American states and provinces (Table 1, Fig 1). From these data, we selected nine life history variables expected to vary geographically and influence population growth: 1) adult male snout-vent length (SVL), 2) adult female SVL, 3) proportion of gravid females, 4) litter size, 5) maternal SVL–litter size relationship (hereafter, size–fecundity relationship), 6) neonate SVL, 7) neonate mass, 8) age-zero annual growth (growth prior to first hibernation), and 9) age-one annual growth (growth between first and second hibernation). Data meeting our sample size criteria for these life history traits were available for years 1937–2014 (Table 1). Unless otherwise specified, for each variable we calculated an average for a given study site. Definitions of response variables and sample size criteria for inclusion in the range-wide analyses are described below.

**Table 1:** Sources for Eastern Massasauga life history information including locality and study years.

State/ Province	County/ District/ Municipality	Year(s)	Life history variable(s)	Source citation or co-author initials
IA	Bremer	**2002–2012**	**1**, **2**, 3, **4**, **6–9**	TJV
IL	Clinton	**1999–2011**	**1–9**	[19, 29–33], MJD, CAP, SJW
IL	DuPage	1941	5, 6	[34]
IL	*Cook/ Lake	**1937–1938; 1982–1991;1999–2009**	2, 3, **4**, 6, 7	[35–38], TGA, MR
IL	Piatt	**2002–2006**	1, 2, **4**, 5, **6**	CAP
IL	Warren	1971–1972	1, 2, 5, 6, 7	[39]
IL	Will	1992–1994	1, 2	DM; TGA
IN	Hendricks	1886	5	[40]
IN	LaGrange	**1999–2001**	**1**, 2, **4**, **6**, **7**, **9**	[41], BAK, JCM
IN	Marshall	1959	5, 6	[42]
MI	Barry	**2004–2013**	**1–5**, 6, 7, **9**	[43, 44], RLB, KMB, DRB, RC, BDK, JAM
MI	Cass	**2006–2014**	**1–9**	[45–47], MDC; LJF; ETH
MI	Kalkaska	**2002–2007**	**1**, **2**, **4–7**	[48–52], NDB, BAD, BAK, JCM, JMR
MI	Lenawee	**2003–2004**	1, **2**	[53, 54], JAM
MI	Oakland	2002–2006	2	[55, 56], BAK
MI	Van Buren	2004–2008	1, 2	MDC, JAM
MI	Washtenaw	**2010–2011**	1, **2**, 3	TGA, GSC, DM, YL
NY	Genesee	**2006–2013**	1, **2**, 3, **4**, **6**, **7**	[57], JMA, KTS
NY	Onondaga	**1988–1993; 2006–2013**	**1–4**, **6–8**	[57–59], TB, BDJ, GJ, KTS
OH	Ashtabula	**2002–2003**	1, 2, **4**, **6–9**	GL; DW
OH	Champaign	2007	1, 2, 6, 7	JGD
OH	Clark	2007	1, 2, 6, 7	JGD
OH	Greene	1993	5–7	[60]
OH	*Greene / Warren	2003–2007	1, 2	JGD
OH	Hardin	1931	5, 6	[61, 62]
OH	Wyandot	**1994–2012**	**1**, **2**, **4–9**	DW
ONT	Bruce	**2001–2004**	**1**, **2**, **4**, **6**, **7**	[63–69], DSH
ONT	Essex	**2000–2004**	1, 2, **4**, **6**, **7**	KC, AML, PDP, TP
ONT	Muskoka	**2002–2009**	**2–9**	JDR
ONT	Parry Sound Beausoliel Island	**1978–2008**	**1, 2**	[7, 70], MD, AP, KAP, SS
ONT	Parry Sound	**1995–1996**	**4, 6, 7**	[71, 72]
		**1992–2009**	**1, 2, 4–8**	[73], JDR
ONT	Niagara	**2000–2012**	**1**, **2**, 3, **4–9**	AY
PA	*Butler/ Venango	1977; 1933; 2003–2013	**1–9**	[74–76], BCJ, MJK, HKR
WI	Buffalo	**1967; 2000–2007**	**1–5**, 6, **7**, **9**	[77–79], RWH, RAP
WI	*Juneau/ Monroe	1994–2004 1999–2000	7	[80, 81]

Due to the sensitive nature of locality data, study site information is reported at the county, district, or municipality level. Datasets with life history variables meeting sample size criteria are bolded. Notation for life history variables: 1, adult male SVL; 2, adult female SVL; 3, proportion gravid; 4, litter size; 5, size-fecundity relationship; 6, neonate SVL; 7, neonate mass; 8, age-zero growth; 9, age-one growth

*, data pooled due to close proximity between study sites.

### Response variables

#### Adult SVL

We defined adult male SVL and adult female SVL as the mean SVL of the ten largest individuals for each sex, provided data were available for at least 20 individuals ≥ 40 cm SVL of a given sex per study site. To ensure that including sites with moderate sample sizes (20–44 individuals, N = 8) with sites that had large sample sizes (≥ 95 individuals per site, N = 9) did not significantly influence our results [82, 83], we also computed estimates from the top-ranked model (see Modeling section below) using only sites with large sample sizes. The overall pattern remained unchanged and confidence intervals broadly overlapped between the two models.

#### Proportion gravid

We estimated the proportion of gravid females as the ratio of gravid females to the total number of adult females captured, provided N ≥ 50 adult females captured per study site. Where data permitted, we calculated a weighted average by year for a given site. Reproductive status was determined by detection of enlarged follicles via palpation, x-ray, or ultrasound. Due to insufficient recaptures across study sites, estimates are unadjusted for differences in detection probabilities between gravid and non-gravid females. As gravid females have higher detection probabilities than non-gravid females (E. T. Hileman, unpublished data), we anticipated these estimates would be positively biased [84]. If the magnitude of this bias can be assumed to be similar across study sites, then any differences detected among study sites should be biologically meaningful even if point estimates are biased.

#### Litter size

We defined litter size as the mean number of captive offspring produced per wild-caught female, including stillborns, provided N ≥ 3 litters per study site.

#### Size–fecundity relationship

We characterized the size–fecundity relationship across sites with N ≥ 8 litters using analysis of covariance with litter size as the response variable, study site as a fixed factor, and maternal SVL as a covariate. Litter size is expected to increase as a function of maternal abdominal volume, which is a cubic function of maternal length [85]. Therefore, we natural log-transformed litter size and SVL prior to analysis. This relationship did not differ in slope (site-by-maternal SVL interaction, *F*_9, 204_ = 0.44, *P* = 0.91) but did differ in elevation among sites (site, *F*_1, 213_ = 37.32, *P* < 0.0001). Therefore, to compare sites, we computed the expected litter size of an average adult female Eastern Massasauga (SVL = 55.2 cm, mean size of adult females from Cass County, Michigan). We used mean female size from the Cass County population due to its proximity to the range center for the species. In other words, we assumed that an adult female SVL of 55.2 cm falls within the size range of adult females across the range.

#### Neonate SVL and mass

Neonate SVL and mass represent average measurements at birth, excluding stillborns, provided N ≥ 3 litters or ≥ 25 neonates per study site.

#### Age-zero and age-one growth

**Fig 2:**
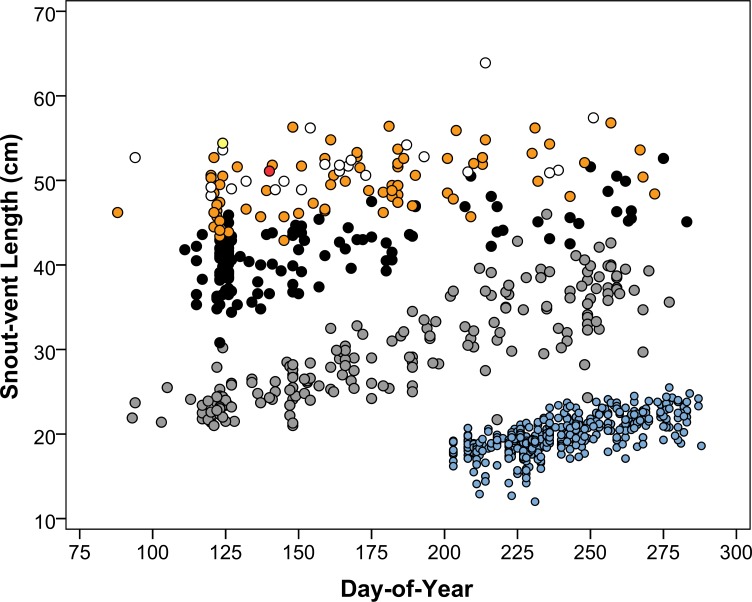
Snout‐vent length plotted against capture day‐of‐year of Eastern Massasaugas captured during 2006–2014 in Cass County, Michigan. Seven distinct age classes are evident: age 0 (blue) includes recently born animals prior to their first hibernation; age 1 (gray) includes animals captured following their first hibernation, but prior to their second hibernation; age 2 (black) includes animals captured following their second hibernation, but prior to their third hibernation, and so on; age 3 (white); age 4 (orange); age 5 (red); age 6 (yellow). Age 1 begins to overlap with age 2 in August (~ DOY 225).

For age-zero growth and age-one growth, we first plotted SVL against capture day‐of‐year (DOY). Visualized graphically, individuals captured in their birth year (age-zero) are identifiable as distinct clusters (Fig 2, S1 File). Similarly, individuals that survived their first winter but have not experienced a second winter (age-one) are identifiable early in the season. However, later in the season some age-one and older individuals begin to overlap in size (Fig 2). In these instances, age assignment was somewhat arbitrary but necessary to obtain an adequate sample size. For age-zero we required N ≥ 35 captures over a time span ≥ 46 days per study site; for age-one we required N ≥11 captures over a time span ≥ 106 days per study site. Age-zero individuals are born in summer and thus have a shorter capture time span due to the truncated active season. Analysis of covariance with SVL as the response variable, study site as a fixed factor, and DOY as a covariate revealed significant differences in daily growth rate among sites (site-by-DOY interaction for age-zero, *F*_8, 1344_ = 5.77, *P* < 0.0001; for age-one, *F*_10, 608_ = 3.78, *P* < 0.0001).

Because growing season length varies across sites, we multiplied daily growth rates by the average number of ‘growing days’ to obtain annual growth rates for age-zero and age-one. We defined growing days for a given site as the number of days (averaged over ≥ 15 years) the minimum average daily temperature exceeded 5°C. For age-zero, we included growing days from the estimated birth date (date of first neonate capture) through fall. For age-one, we included growing days from spring through fall. For sites in the United States, we used 1981–2010 daily climate normals from the National Oceanic and Atmospheric Administration [86]. For sites in Canada, we used 1965–2015 daily almanac averages and extremes from Environment Canada (http://climate.weather.gc.ca/climate_normals/index_e.html). Details on weather station selection are provided below.

### Explanatory variables

Study site Cartesian coordinates were provided by collaborators or approximated using published locality information and Google Earth (version 7.1.5.1557). We used these coordinates to identify the nearest weather data logging station to each study site (mean distance = 16.67 km ± 12.13 SD) using web tools from the National Oceanic and Atmospheric Administration (NOAA, http://www.ncdc.noaa.gov/cdo-web/datatools/normals), Midwestern Regional Climate Center (Cli-MATE, http://mrcc.isws.illinois.edu/CLIMATE/), and Environment Canada (EC, http://climate.weather.gc.ca/climate_normals/index_e.html). We acquired 30-year annual climate normals (1981–2010) from these websites and considered only climate variables that we *a priori* hypothesized would have strong explanatory power and were available for sites in the United States and Canada. In addition to latitude and longitude, these included the 30-year annual climate normals for mean temperature (°C, MT), mean precipitation (mm, MP), frost-free days (FFD), and growing degree-days (GDD). MP includes rainfall plus snow water equivalent. The number of FFD is based on a 90% probability that the last spring frost did not occur earlier in the season and the first fall frost did not occur later in the season. GDD is the sum of degrees by which daily mean temperature is above 10°C.

### Modeling

To elucidate geographic and climatic patterns of variation in life history, we used general linear models and ordinary least square methods with geographic coordinates and the four climate normals as explanatory variables. To address scaling differences, we z-transformed explanatory variables before analysis to make model effect sizes (slopes) comparable within a given candidate set. Ninety-five percent confidence intervals for coefficients were calculated using the *t*-distribution. We employed multimodel inference using an information-theoretic approach and Akaike’s information criterion adjusted for small sample size (AIC_c_, [87, 88]).

We avoided including over-fitted models in our candidate sets by requiring ≥ 7 observations per explanatory variable [89]. For analyses to be considered confirmatory rather than exploratory, the number of models (*R*) considered in a candidate set should be far fewer than the number of observations (*N*, [88, 90]). We achieved this by restricting the number of candidate models per analysis to $R\leq \frac{1}{2}N$. As anticipated, latitude, frost-free days, growing degree-days, and mean temperature were highly correlated with one another (Pearson’s correlation coefficient = 0.82–0.96). We avoided issues associated with multicollinearity by restricting use of these predictors to separate models (i.e., we considered only models that included latitude or frost-free days or growing degree-days or mean temperature singly or in combination with longitude and mean annual precipitation). We did not correct for spatial autocorrelation using partial Mantel tests [91, 92] as these methods have been shown to be problematic and can yield biased results [93].

To aid in interpretability of results across analyses, latitude, mean annual precipitation, mean annual temperature, and frost-free days were included in all candidate sets. Response variables with larger sample sizes (N ≥ 10) included additional models (Table 2). We performed general linear model analyses and multimodel inference using R [94] and the package ‘AICcmodavg’ [95]. We used the R package ‘ggplot2’ [96] to graphically depict relationships between untransformed explanatory and response variables for the best supported candidate models based on AIC_c_ weights. Since candidate model sets were non-nested and therefore lacked a global model, we evaluated the top-ranked model for each candidate set using residual analyses consisting of Q-Q plot and Cook’s distance to ensure the assumption of normality was adequately met.

**Table 2:** (A) Number of study sites, number of explanatory variables per model, and number of candidate models possible and (B) specific candidate models used in analyses of Eastern Massasauga life history response variables.

A. Number of study sites, number of explanatory variables per model, and number of candidate models.										
		Life history response variables								
Criteria		Adult Male SVL (cm)	Adult Female SVL (cm)	Proportion gravid	Litter size	Size–fecundity relationship	Neonate SVL (cm)	Neonate mass (g)	Age-zero Growth (cm/year)	Age-one Growth (cm/year)
Number of study sites	*N*	14	17	8	20	10	17	18	9	11
Number of explanatory variables per model	*K*	2	2	1	2	1	2	2	1	1
Number of candidate models per analysis	*R*	7	8	4	10	5	8	9	4	5
B. Specific candidate models used for each analysis.										
	1)	LAT	LAT	LAT	LAT	LAT	LAT	LAT	LAT	LAT
	2)	MT	MT	MT	MT	MT	MT	MT	MT	MT
	3)	MP	MP	MP	MP	MP	MP	MP	MP	MP
	4)	FFD	FFD	FFD	FFD	FFD	FFD	FFD	FFD	FFD
	5)	GDD	GDD		GDD	GDD	GDD	GDD		GDD
	6)	LONG	LONG		LONG		LONG	LONG		
	7)	MP+FFD	MP+FFD		MP+FFD		MP+FFD	MP+FFD		
	8)		MP+MT		MP+MT		MP+MT	MP+MT		
	9)				MP+GDD			MP+GDD		
	10)				MP+LAT					

Shaded region represents four models used for all variables. Abbreviations: LAT–Latitude, LONG–Longitude, FFD–Frost-free days, GDD–Growing degree-days, MP–Mean Precipitation, MT–Mean Temperature

+ denotes additive effects.

## Results

Data meeting our sample size criteria were available for 8–20 Eastern Massasauga study sites depending on the variable, allowing inclusion of one or two explanatory variables per model and analysis of 4–10 models per variable (Table 2, S2 File). Of the nine life history variables included, six (described individually below) provided evidence for an association with one or more explanatory variables, whereas three (adult male SVL, neonate SVL, age 1 growth) did not (Table 3). The untransformed effect size, 95% confidence intervals, and equation of the line are provided below for the top-ranked model of each candidate set from Table 3.

**Table 3:** Candidate model sets for life history variables. Bolded 95% confidence intervals exclude zero and therefore indicate the standardized effect size for a given explanatory variable is informative. For models with two predictor variables, the standardized effect size and 95% CI for the first and second variable are in the first and second row associated with that model. Model abbreviations are the same as in Table 2.

	*Model*	*K*	*AIC*_*c*_	*ΔAIC*_*c*_	*W*_*i*_	*Log(*L*)*	*R*^*2*^	*Effect Size*	*95% CI*
*Adult male SVL*									
1)	MP	3	91.48	0.00	0.36	-41.54	0.17	2.14	-0.83–5.12
2)	LAT	3	93.25	1.77	0.15	-42.43	0.06	1.21	-1.85–4.26
3)	MT	3	93.53	2.05	0.13	-42.56	0.04	-1.01	-4.16–2.13
4)	FFD	3	93.91	2.43	0.11	-42.76	0.01	-0.59	-3.89–2.71
5)	GDD	3	94.04	2.56	0.10	-42.82	0.00	-0.31	-3.48–2.86
6)	Long	3	94.04	2.56	0.10	-42.82	0.00	-0.25	-2.96–2.46
7)	MP + FFD	4	95.46	3.98	0.05	-41.51	0.17	2.10	-1.06–5.26
								-0.32	-3.53–2.9
*Adult female SVL*									
1)	MP	3	97.92	0.00	0.69	-45.04	0.54	3.34	**1.65–5.03**
2)	MP + FFD	4	100.87	2.95	0.16	-44.77	0.56	3.09	**1.20–4.99**
								-0.62	-2.60–1.36
3)	MP + MT	4	101.07	3.16	0.14	-44.87	0.55	3.22	**1.41–5.03**
								-0.48	-2.46–1.50
4)	FFD	3	108.05	10.13	0.00	-50.10	0.17	-1.95	-4.32–0.42
5)	MT	3	109.74	11.82	0.00	-50.95	0.08	-1.43	-4.05–1.19
6)	GDD	3	110.68	12.76	0.00	-51.41	0.03	-0.89	-3.64–1.85
7)	LAT	3	110.83	12.91	0.00	-51.49	0.02	0.77	-2.04–3.58
8)	LONG	3	110.89	12.97	0.00	-51.52	0.02	0.60	-1.79–2.99
*Proportion of gravid females*									
1)	LAT	3	-2.34	0.00	0.85	7.17	0.68	0.12	**0.04–0.21**
2)	MT	3	1.95	4.29	0.10	5.03	0.45	-0.10	-0.20–0.01
3)	FFD	3	4.18	6.52	0.03	3.91	0.27	-0.07	-0.19–0.05
4)	MP	3	5.70	8.04	0.02	3.15	0.11	-0.05	-0.18–0.09
*Litter size*									
1)	MT	3	94.30	0.00	0.32	-43.40	0.26	-1.22	**-2.24 –-0.20**
2)	FFD	3	95.06	0.77	0.22	-43.78	0.23	-1.08	**-2.06 –-0.10**
3)	LAT	3	95.32	1.02	0.19	-43.91	0.22	1.15	**0.08–2.21**
4)	MP + MT	4	97.43	3.13	0.07	-43.38	0.26	-0.08	-1.13–0.97
								-1.25	**-2.36 –-0.13**
5)	GDD	3	97.63	3.33	0.06	-45.06	0.12	-0.87	-2.01–0.27
6)	MP + FFD	4	97.98	3.68	0.05	-43.66	0.24	-0.25	-1.38–0.88
								-1.19	**-2.32 –-0.06**
7)	MP + LAT	4	98.35	4.05	0.04	-43.84	0.23	0.17	-0.86–1.19
								1.12	**0.02–2.23**
8)	MP	3	99.95	5.65	0.02	-46.22	0.02	0.30	-0.81–1.41
9)	Long	3	100.04	5.74	0.02	-46.27	0.01	-0.24	-1.30–0.82
10)	MP + GDD	4	100.79	6.49	0.01	-45.06	0.13	0.03	-1.11–1.18
								-0.86	-2.11–0.39
*Size–fecundity relationship*									
1)	LAT	3	-2.93	0.00	0.58	6.47	0.56	0.13	**0.04–0.22**
2)	MT	3	-0.98	1.95	0.22	5.49	0.47	-0.11	**-0.21 –-0.01**
3)	GDD	3	0.53	3.46	0.10	4.73	0.38	-0.10	-0.21–0.00
4)	FFD	3	1.08	4.01	0.08	4.46	0.35	-0.09	-0.19–0.01
5)	MP	3	4.31	7.24	0.02	2.85	0.10	-0.05	-0.17–0.07
*Neonate SVL*									
1)	Long	3	49.18	0.00	0.29	-20.67	0.17	-0.34	-0.76–0.08
2)	MP	3	50.22	1.04	0.17	-21.19	0.12	0.29	-0.16–0.74
3)	LAT	3	50.64	1.46	0.14	-21.39	0.09	-0.26	-0.7–0.18
4)	GDD	3	50.98	1.81	0.12	-21.57	0.07	0.05	-0.21–0.67
5)	MP + MT	4	51.64	2.46	0.08	-20.15	0.19	0.14	-0.08–0.96
								0.23	-0.21–0.71
6)	MT	3	51.81	2.64	0.08	-21.98	0.03	0.44	-0.31–0.58
7)	MP + FFD	4	52.15	2.97	0.07	-20.41	0.22	0.25	-0.07–0.88
								0.41	-0.17–0.73
8)	FFD	3	52.22	3.04	0.06	-22.19	0.00	0.28	-0.37–0.48
*Neonate mass*									
1)	MP	3	47.20	0.00	0.59	-19.74	0.44	0.55	**0.22–0.88**
2)	MP + FFD	4	50.02	2.82	0.14	-19.47	0.45	0.59	**0.23–0.94**
								0.11	-0.25–0.48
3)	MP + GDD	4	50.41	3.21	0.12	-19.67	0.45	0.57	**0.21–0.93**
								0.07	-0.35–0.48
4)	MP + MT	4	50.44	3.24	0.12	-19.68	0.44	0.57	**0.21–0.92**
								0.06	-0.33–0.45
5)	LONG	3	54.11	6.91	0.02	-23.20	0.44	0.34	-0.05–0.72
6)	GDD	3	57.29	10.09	0.00	-24.79	0.44	-0.12	-0.63–0.39
7)	MT	3	57.40	10.20	0.00	-24.84	0.44	-0.09	-0.58–0.40
8)	FFD	3	57.46	10.26	0.00	-24.87	0.18	-0.07	-0.52–0.38
9)	LAT	3	57.49	10.29	0.00	-24.89	0.02	0.07	-0.44–0.58
*Age-zero growth*									
1)	LAT	3	43.03	0.00	0.83	-16.11	0.62	-1.94	**-3.30 –-0.58**
2)	MT	3	46.88	3.86	0.12	-18.04	0.42	1.59	-0.09–3.26
3)	FFD	3	49.30	6.27	0.04	-19.25	0.24	1.15	-0.69–3.00
4)	MP	3	51.45	8.42	0.01	-20.33	0.03	-0.47	-2.78–1.84
*Age-one growth*									
1)	MP	3.00	71.53	0.00	0.30	-31.05	0.14	-1.66	-4.77–1.45
2)	FFD	3.00	71.91	0.38	0.25	-31.24	0.11	1.56	-1.81–4.93
3)	MT	3.00	72.62	1.09	0.17	-31.60	0.05	1.06	-2.45–4.58
4)	GDD	3.00	72.92	1.39	0.15	-31.75	0.00	-0.04	-3.55–3.47
5)	LAT	3.00	73.17	1.64	0.13	-31.87	0.02	0.73	-2.89–4.36

**AIC**_**c**_ is Akaike’s information criterion adjusted for small sample size. **ΔAIC**_**c**_ is the difference between the **AIC**_**c**_ of model *i* and that of the top-ranking model. AIC_c_ weight (**W**_***i***_) is the probability that model *i* is the best model given the data and the other hypothesized models in the candidate set. AIC_c_ weights must sum to 1. **K** is the number of parameters in the model (i.e., intercept, slope(s), and residual variance). ***Log(***L***)*** is the natural logarithm of the likelihood function.

### Adult female SVL

**Fig 3:**
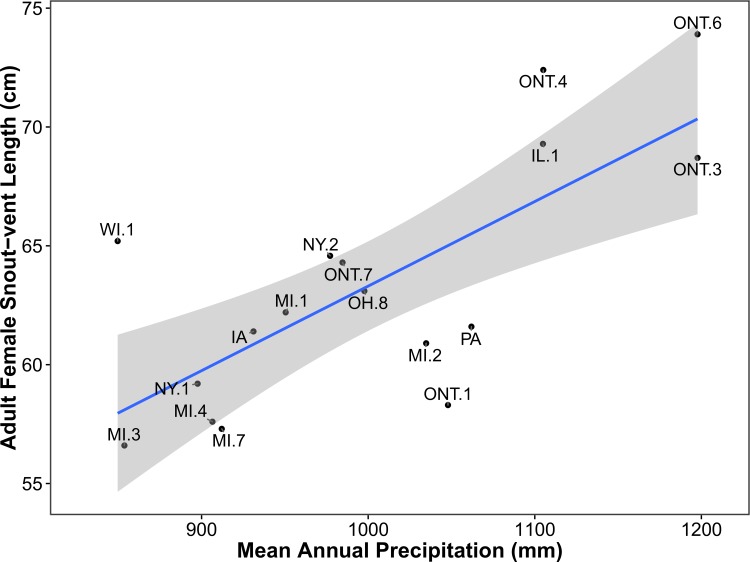
Relationship between mean annual precipitation (untransformed) and adult female size as explained by the top-ranked model using AIC_c_ (Table 3). The shaded area represents the smoothed 95% CI using t-based approximations. County and district abbreviations are as in Fig 1.

–Among 17 sites, adult female mean SVL ranged from 56.6 cm in Kalkaska County, Michigan, to 73.9 cm in the Parry Sound District, Ontario, with an overall mean of 63.3 cm. Of the eight models used to explain geographic variation in adult female SVL, the top three received 99% of the support based on AIC_c_ weights (Table 3). All three of these models included mean precipitation (MP) as an explanatory variable and in all three MP was informative as indicated by 95% confidence intervals (CI) for effect size that excluded zero. Models two and three were simple additive embellishments of model MP which included the uninformative (i.e., 95% CI for effect sizes included zero) variables frost-free days and mean temperature, respectively (Table 3). These lower ranking models cannibalized 0.30 of the AIC_c_ weight which would have otherwise been absorbed by the top-ranked model. Based on the top-ranked model (Table 3, Fig 3), adult female SVL increased by 3.6 cm (95% CI = 1.8–5.4) for every 100 mm increase in mean annual precipitation (Y = 0.03558X + 27.73).

### Proportion gravid

**Fig 4:**
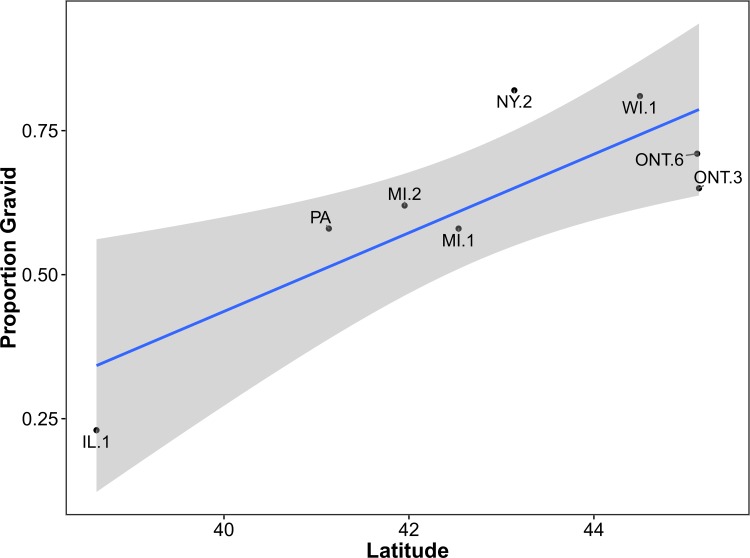
Relationship between latitude (untransformed) and the proportion of gravid females as explained by the top-ranked model using AIC_c_ (Table 3). The shaded area represents the smoothed 95% CI using t-based approximations. County and district abbreviations are as in Fig 1.

–Among eight sites, the proportion of gravid females ranged from 23% in Clinton County, Illinois, to 82% in Onondaga, New York, with an overall mean of 62%. Latitude was strongly supported (*W*_*i*_ = 0.85) as an explanatory variable for the proportion of gravid females for a given site with the proportion of gravid females increasing with increasing latitude (Table 3, Fig 4). Latitude was the only explanatory variable with an effect size differing significantly from zero. Mean temperature, frost-free days, and mean precipitation were all considered uninformative because confidence intervals included zero. Based on the top-ranked model (Table 3, Fig 4), the proportion of gravid females increased 0.07 (95% CI = 0.02–0.12) for every 1°increase in latitude (Y = 0.0682X + –2.29).

### Litter size

**Fig 5:**
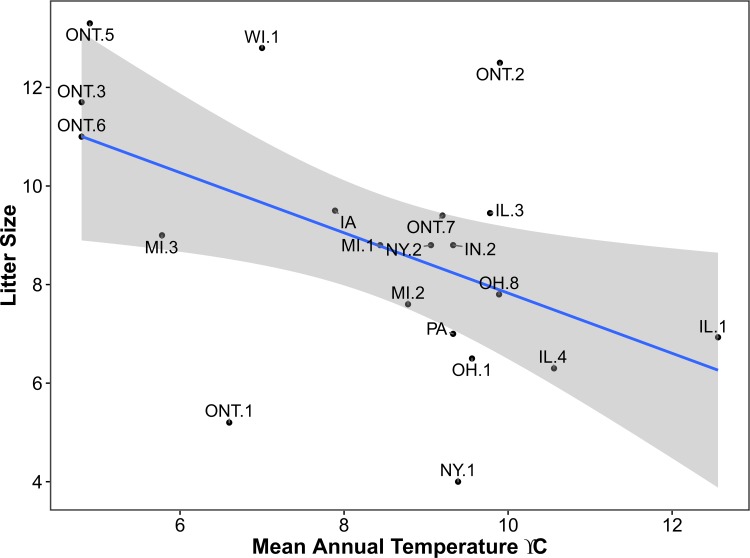
Relationship between mean annual temperature (untransformed) and litter size as explained by the top-ranked model using AIC_c_ (Table 3). The shaded area represents the smoothed 95% CI using t-based approximations. County and district abbreviations are as in Fig 1.

–Among 20 sites, mean litter size ranged from 4.0 in Genesee County, New York, to 13.3 in the Parry Sound District, Ontario, with an overall mean of 8.8. Mean temperature had a negative effect size and received modest support (*W*_*i*_ = 0.32) as an explanatory variable for litter size (Table 3, Fig 5), as did models including frost-free days (*W*_*i*_ = 0.22) and latitude (*W*_*i*_ = 0.19), reflecting strong positive correlations among these explanatory variables. Additive models (4, 6, and 7) were weakly supported (*W*_*i*_ = 0.04–0.07) due to the presence of informative explanatory variables mean temperature, latitude, or frost-free days. Mean precipitation had an effect size that included zero and was thus considered uninformative. Based on the top-ranked model (Table 3, Fig 5), litter size decreased by one neonate (95% CI = 0.2–1.8) for every 1.64°C increase in mean annual temperature (Y = –0.6111X + 13.94).

### Size–fecundity relationship

**Fig 6:**
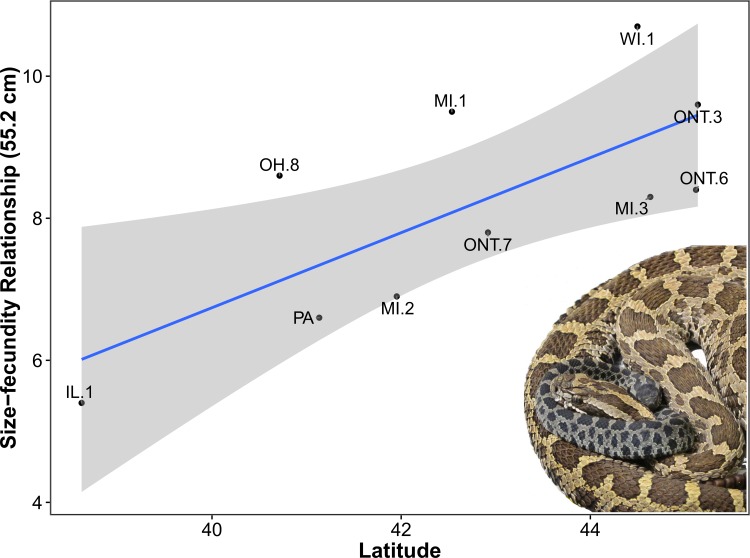
Relationship between latitude (untransformed) and size–fecundity (natural log back-transformed) as explained by the top-ranked model using AIC_c_ (Table 3). Female size was held constant at 55.2 cm SVL based on the average size of adult females in Cass County, Michigan. The shaded area represents the smoothed 95% CI using t-based approximations. County and district abbreviations are as in Fig 1. The image of dam and offspring was taken within minutes of parturition in Cass County, Michigan (Photograph credit, E. T. Hileman).

–Among 10 sites, expected litter size for a typical female (SVL = 55.2 cm) ranged from 5.4 in Clinton County, Illinois, to 10.7 in Buffalo County, Wisconsin, with an overall mean of 8.2. Latitude was supported (*W*_*i*_ = 0.58) as an explanatory variable of the size–fecundity relationship (Table 3, Fig 6). Latitude also had the largest and only positive effect size. Mean temperature also garnered some support (*W*_*i*_ = 0.22). The remaining three models included the uninformative variables growing degree-days, frost-free days, or mean precipitation. Based on the top-ranked model (Table 3, Fig 6), expected litter size of a typical female increased by one neonate (95% CI = 0.2–1.8) for every 1.89°increase in latitude (Y = 0.5280X + –14.38).

### Neonate mass

**Fig 7:**
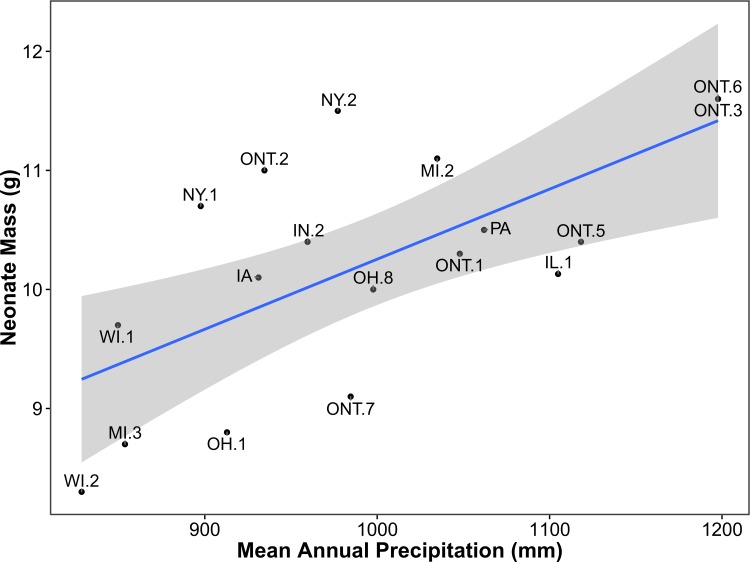
Relationship between mean annual precipitation (untransformed) and neonate mass as explained by the top-ranked model using AIC_c_ (Table 3). The shaded area represents the smoothed 95% CI using t-based approximations. County and district abbreviations are as in Fig 1.

Among 18 sites, mean neonate mass ranged from 8.3 g in Juneau and Monroe Counties, Wisconsin, to 11.6 g in the Muskoka District, Ontario, with an overall mean of 10.2 g. Models including the explanatory variable mean annual precipitation had the largest (positive) effect sizes and best explained geographic variation in neonate mass (Table 3, Fig 7). The top four of nine models were considered informative due to the inclusion of the explanatory variable mean precipitation. Similar to the first candidate model set (adult female size), models 2–4 were embellishments of model one that included the uninformative additive effects of frost-free days, growing degree-days, and mean temperature, respectively. These models cannibalized 0.38 of the AIC_c_ weight which would have otherwise been allotted to model MP (*W*_*i*_ = 0.59). Based on the top-ranked model, neonate mass increased by 0.6 g (95% CI = 0.2–0.9) for every 100 mm increase in mean annual precipitation (Y = 0.00589X + 4.37).

### Age-zero growth

**Fig 8:**
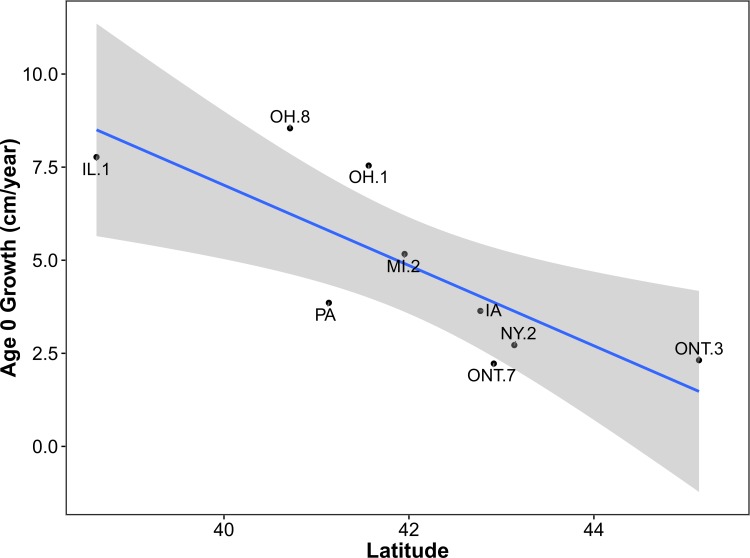
Relationship between latitude (untransformed) and age-zero annual growth as explained by the top-ranked model using AIC_c_ (Table 3). The shaded area represents the smoothed 95% CI using t-based approximations. County and district abbreviations are as in Fig 1.

Among nine sites, age-zero growth ranged from 2.2 cm/year in the Regional Municipality of Niagara, Ontario, to 8.5 cm/year in Clinton County, Illinois, with an overall mean of 5.1 cm/year. Latitude was strongly supported (*W*_*i*_ = 0.83) as an explanatory variable for age-zero growth (Table 3, Fig 8). It also had the largest (negative) effect size. Mean temperature, frost-free days, and mean precipitation were all considered uninformative because confidence intervals included zero. Based on the top-ranked model (Table 3, Fig 8), annual growth decreased 1.1 cm (95% CI = 0.3–1.8) for every 1°increase in latitude (Y = –1.0783X + 50.1489).

## Discussion

Our study is one of few to investigate intraspecific variation in squamate life history traits over the geographic extent of a species’ range (e.g., [6–8, 91]). Others have investigated intraspecific variation in reptile life history traits at local ecotypic [9, 10] or broader but incomplete geographic scales [15, 97–102]. Our range-wide analyses provide strong evidence for geographic patterns in six of the nine examined Eastern Massasauga life history traits. Adult female SVL and neonate mass increased with increasing mean annual precipitation (Table 3). Litter size decreased with increasing mean temperature, and the size–fecundity relationship and age-zero growth both increased with increasing latitude. The proportion of gravid females also increased with increasing latitude, but as discussed below, this may be the result of geographically varying detection bias in gravid and non-gravid females. We did not find evidence for geographic variation in adult male SVL, neonate SVL, or age-one growth (Table 3). However, this is likely due to a combination of small sample sizes (i.e., lack of power) and measurement error rather than the absence of geographic variation in these life history traits. For example, for age-one growth, some individuals captured late in the active season may have been erroneously included or excluded due to size overlap between age class one and older individuals, impeding our ability to detect a geographic pattern (Fig 2).

Body size impacts key aspects of an organism such as physiology, survival, fecundity, and longevity, making it one of the most important life history traits ([16, 103–105]. The tendency for body size to be larger in cooler environments is described by Bergmann’s rule [106] and the temperature-size rule [107], which collectively represent the most taxonomically widespread rules in biology (summarized in [4]). Bergmann’s rule describes variation in body size that may include both plastic responses and heritable traits, whereas the temperature-size rule describes variation in body size attributable to phenotypic plasticity [4]. Examples of this general pattern of variation in body size abound in endotherms [108–110] and ectotherms [111–113], but there are exceptions (e.g., [114, 115]). In reptiles, using latitude or temperature as proxies, chelonians followed Bergmann’s rule (33 of 38 species), whereas the majority of squamates (101 of 139 reviewed) followed its inverse (i.e., larger body sizes in warmer environments) [116].

In our analysis, mean precipitation best explained variation in adult female SVL rather than latitude or mean temperature (as in Bergmann’s rule, or its inverse) or one of their correlates (e.g., frost-free days, growing degree-days). However, given the distribution of the Eastern Massasauga and influence the Great Lakes have on local climate, it is not entirely surprising that females are larger where there is more precipitation. Westerly winds accumulate moisture as they move across the Great Lakes, resulting in snow belts and higher rates of annual precipitation principally off the eastern and southern shores [14]. Precipitation directly contributes to primary productivity and thus may influence prey availability and abundance [10, 97, 117–120]. In European Grass Snakes (*Natrix natrix*), females of a mainland population were significantly larger than those of an island population due to the presence of larger prey (mainly toads) on the mainland [121]. Based on fecal analyses, Eastern Massasauga populations in Ontario and Ohio overlapped in 5 of 13 prey species [69]. However, snakes that consumed smaller versus larger prey animals did not differ significantly in size in Ontario or Ohio [69]. Whether prey species differ between other populations enough to impact adult body size is unknown [33, 122, 123]. A stronger influence on variation in adult body size may be the duration that prey and water are available to massasaugas during their active season [10]. Larger SVLs were associated with higher precipitation in four lacertid species (*Phoenicolacerta laevis*, *Ophisops elegans*, *Acanthodactylus boskianus*, and *Mesalina guttulata*, [124]). Similarly, body size in the Eastern Side-blotched Lizard (*Uta stansburiana stejnegeri*) was significantly larger in a wet year when compared to a dry year [125]. Conversely, in frog-eating snakes (*Tropidonophis mairii*), temporal variation in rainfall did not affect adult female body length but did increase prey abundance and snake clutch size [119].

Our results suggest that multiple factors influence geographic variation in reproductive output. We found evidence for a negative relationship between litter size and mean annual temperature such that litter size is larger at cooler (more northerly) sites, thus corroborating the results of Aldridge et al. (2008). This latitudinal pattern is not solely due to variation in maternal size: when holding female size constant (55.2 cm, SVL; ANCOVA), litter size still increased one neonate (95% CI = 0.2–1.8) for every 1.89°increase in latitude. Thus, litter size increases because females are generally larger in the north, especially where mean precipitation is higher, and because the female size-specific fecundity is greater. Furthermore, neonate mass increased with increasing mean precipitation, indicating that at those northerly sites where precipitation is high, females produce larger litters and heavier offspring.

Geographic variation in sexual maturity and reproductive frequency may help explain size-specific fecundity. Eastern Massasaugas reach sexual maturity between 2–3 years old at the southern extent of their range (Clinton Co., IL, [19]) and 4–6 years old at the northern extent (the Parry Sound District, Ontario, Canada, J. D. Rouse, unpublished data). On average, there are 108 fewer frost-free days in the Parry Sound District than in Clinton County (NOAA, EC). The shorter growing season in northern latitudes is likely an important contributor to geographic variation in the age of sexual maturity [19, 120]. In support of this, we found evidence for latitudinal variation in annual growth rates for age-zero, with fast growers in the south and slow growers in the north (a decrease of 1.1 cm, 95% CI = 0.3–1.8, for every 1°increase in latitude). Delayed sexual maturity in northern latitudes reduces reproductive potential. However, increased survival [7], litter size ([19, 120], this study), and longevity [126] may serve as compensatory mechanisms to overcome this reduction in reproductive potential. Based on our results, maternal size alone cannot adequately explain the increase in litter size with increasing latitude. An alternative explanation is that lower reproductive frequencies in the north may provide females time to accumulate the necessary energetic capital to increase litter size without a concomitant increase in female body size. Because active season length decreases along a latitudinal gradient, reproductive frequencies in northern populations are predicted to decrease with increasing latitude [120, 127]. Based on the proportion of gravid females, Eastern Massasaugas may generally reproduce biennially in Illinois [19], Indiana (J. C. Marshall, pers comm), Wisconsin (R. W. Hay, pers comm, but see [77] for a report of annual reproduction), Michigan (E. T. Hileman, unpublished data), and Pennsylvania [75]. However, reproductive frequencies for populations in northern latitudes are largely unknown.

Of the life history traits we analyzed, only the results from the proportion of gravid females analysis yielded results contrary to previous predictions [120, 127]. The estimated proportion of gravid females increased 0.07 (95% = 0.02–0.12) for every 1°increase in latitude. If females in the north require more time to recover from post-partum depletion of fat stores than females in the south, it should be reflected in a negative relationship between the proportion gravid and latitude. Markedly lower reproductive frequencies have been recorded in Timber Rattlesnakes near the northern extent of their range [128, 129]. In the Australian elapid (*Drysdalia coronoides*), females reproduce annually in warmer climates and bi- or triennially in the coldest climates [130]. In our experience, the thermoregulatory behavior of gravid Eastern Massasaugas (e.g., [43]) increases their detectability compared to non-gravid females (E. T. Hileman, unpublished data). In using proportion gravid as a surrogate for reproductive frequency, we had assumed the bias in detection probability was constant across sites. This may not be the case given the heterogeneity of Eastern Massasauga habitats across their range (E. T. Hileman, pers obs) and variation in sampling across sites. Viviparity buffers developing embryos from unfavorable thermal conditions, thus allowing viviparous species to live in colder climates than oviparous species [131, 132]. However, given the colder active-season temperatures in the north, gravid females may need to bask more frequently during gestation to maintain optimal embryonic temperatures and reduce the incidence of stillborns [133]. Additionally, parturition must occur early enough to permit neonates adequate time to locate suitable hibernacula. Consequently, the apparent increase in the proportion of gravid females with latitude may be an artifact of differences in behavior and detection probabilities of gravid females across sites. Ideally, multistate models could be used to account for differences in detection probability between gravid and non-gravid females and across sites [134]. Unfortunately, these models are data hungry and require multiple years of capture-recapture data, which will preclude their use for all but the largest of datasets (but see [135] for an example with Meadow Viper, *Vipera ursinii ursinii*, using a 28-year capture-recapture dataset).

Reproductive frequency may be the most important but poorly understood aspect of reproductive biology in snakes [18, 136]. Whether reproductive frequency is annual, biennial, triennial, or less frequent, it is likely driven by age structure, thermal regulation opportunities, and food availability [128, 129, 137–139]. Eastern Massasaugas are longer lived in the northeast than in the southwest [7]. Thus, at higher latitudes age structure is likely skewed toward older adults, with the largest individuals in areas with the highest precipitation. Reproductive frequency may be higher in populations that are skewed toward larger adults in Eastern Cottonmouths (*Agkistrodon piscivorus* [138]). As evidence of this, larger individuals were more likely to be gravid and possessed higher levels of lipid reserves than smaller adults [138, 140, 141]. Food availability in turn is regulated, in part, by precipitation rates, season length, and the availability of appropriate vegetation types to support prey populations. Due to the shorter active season at higher latitudes, massasauga females in the north may need additional time to recover fat stores lost during gestation and parturition and thus may reproduce less frequently than those in the south. Because they are capital breeders, lowered reproductive frequencies should yield proportionally larger energy budgets during years of reproduction, thus resulting in increased litter sizes.

In this study, we provide strong evidence for geographic variation in several Eastern Massasauga life history traits. The degree to which the phenotypic gradients observed here are attributable to plastic responses versus genetic dissimilarities between populations is unknown. Genetic causes for phenotypic differences have been identified in the Western Gartersnake (*Thamnophis elegans*) using common garden experiments with naïve individuals from local mountain meadow or lakeshore habitats [142]. Conversely, Lake Erie Watersnakes (*Nerodia sipedon insularum*) exhibited phenotypically plastic responses to the accidental introduction of a new prey species, the round goby (*Neogobius melanostomus*), via increases to litter and maternal size after the introduction [143, 144]. Previous analyses using microsatellite DNA suggest Eastern Massasauga populations are genetically isolated even over short geographic distances (see Fig 2, [145]). Such isolation might facilitate local adaptation in life history. Conveniently, for predictive modeling, disentangling the product of these two sources of variation is unnecessary.

Climate change is predicted to have a large impact on fauna and flora distributions [146, 147]. Reptiles may be particularly vulnerable due to their thermal constraints and life histories [148]. Thus, understanding climatic variables associated with range-wide variation in life history traits will be useful in predicting life history trait responses to climate change.

Our findings suggest climatic variation in the Great Lakes region influences multiple aspects of Eastern Massasauga life history, including body size, reproduction (litter and offspring size, size-fecundity relationship), and growth. Our results, in combination with information on geographic variation in annual survival [8], will serve conservation efforts by facilitating new iterations of extinction risk models that are biologically more realistic than current models. Of the life history traits we analyzed, size-specific fecundity may be the most important performance measure to consider for future conservation efforts [149], especially if size-based models are used to predict extinction risk. Our analyses also highlight remaining data gaps in Eastern Massasauga life history. Geographic variation in age at maturity and in frequency of reproduction remain poorly documented and these variables can have large effects on population projections. In addition, mechanistic explanations for the associations we document between climatic variables and Eastern Massasauga life history are speculative. A clearer understanding e.g., of the relationship between climatic variables, primary productivity, and prey availability could facilitate Eastern Massasauga conservation through habitat management strategies focused on prey productivity, much as studies of Eastern Massasauga thermal biology have identified habitat management strategies focused on basking habitats [55].

## Supporting information

S1 File Snout-vent length versus day-of-year data. Data used for Fig 2.
(XLSX)

S2 File Range-wide Eastern Massasauga life history data. Data compiled from sources in Table 1. These data were used to create Tables 2 and 3 and Figs 3–8. Due to the Eastern Massasauga’s federally threated status, site specific Cartesian coordinates used in analyses have been redacted.
(XLSX)

S3 File Permission letter for Fig 1. Reprinted and modified from [150] under a CC BY license, with permission from [Collin P. Jaeger], original copyright [2016].
(PDF)
