# Supplementary material for: Climatic and geographic predictors of life history variation in Eastern Massasauga (Sistrurus catenatus): A range-wide synthesis
Source: PLoS One. 2017 Feb 14;12(2):e0172011. doi: 10.1371/journal.pone.0172011 (PMC5308788; doi:10.1371/journal.pone.0172011)
Supplement: S3 File — Reprinted and modified from [150] under a CC BY license, with permission from [Collin P. Jaeger], original copyright [2016]. (PDF) [file pone.0172011.s003.pdf]

Gmail **Eric Hileman <hileman.et@gmail.com>**

Permission request

2 messages

Eric Hileman <ehileman1@niu.edu> Tue, Jan 17, 2017 at 3:19 PM
To: Collin Jaeger <collinjaeger@gmail.com>

Dear Dr. Jaeger:

I request permission for the open-access journal PLOS ONE to publish the following figure (attached) which was modified from figure 1 of your recent publication (listed below) under the Creative Commons Attribution License (CCAL) CC BY 4.0 (http://creativecommons.org/licenses/by/4.0/). Please be aware that this license allows unrestricted use and distribution, even commercially, by third parties. Please reply and provide explicit written permission to publish figure 1 under a CC BY license.

Jaeger CP, Duvall MR, Swanson BJ, Phillips CA, Dreslik MJ, Baker SJ, et al. Microsatellite and major histocompatibility complex variation in an endangered rattlesnake, the Eastern Massasauga (Sistrurus catenatus). Ecology and Evolution. 2016;6(12):3991-4003. doi: 10.1002/ece3.2159. PubMed PMID: WOS:000379342900012.

Thank you!

Eric

--
Eric T. Hileman
Dept. of Biological Sciences
Northern Illinois University
DeKalb, IL 60115

Email: ehileman1@niu.edu
Phone: 779.212.0842

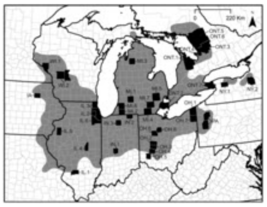

Fig1.tiff
1818K

collinjaeger@gmail.com <collinjaeger@gmail.com> Tue, Jan 17, 2017 at 4:54 PM
To: Eric Hileman <ehileman1@niu.edu>

Permission granted!

Collin
[Quoted text hidden]

<Fig1.tiff>
